# Phase-based treatment versus immediate trauma-focused treatment for post-traumatic stress disorder due to childhood abuse: randomised clinical trial

**DOI:** 10.1192/bjo.2021.1057

**Published:** 2021-11-16

**Authors:** Noortje I. van Vliet, Rafaele J. C. Huntjens, Maarten K. van Dijk, Nathan Bachrach, Marie-Louise Meewisse, Ad de Jongh

**Affiliations:** Dimence Mental Health Group, Deventer, The Netherlands; Department of Experimental Psychotherapy and Psychopathology, University of Groningen, The Netherlands; Dimence Mental Health Group, Deventer, The Netherlands; GGZ Oost Brabant, Boekel, The Netherlands; and Tilburg University, The Netherlands; Abate Center of Expertise in Anxiety and Trauma, The Netherlands; Department of Social Dentistry and Behavioral Sciences, University of Amsterdam and Vrije Universiteit, The Netherlands; and School of Health Sciences, Salford University, Manchester, UK; and Institute of Health and Society, University of Worcester, UK

**Keywords:** Post-traumatic stress disorder, individual psychotherapy, cognitive–behavioural therapies, childhood experience, dissociative disorders

## Abstract

**Background:**

It is unclear whether people with post-traumatic stress disorder (PTSD) and symptoms of complex PTSD due to childhood abuse need a treatment approach different from approaches in the PTSD treatment guidelines.

**Aims:**

To determine whether a phase-based approach is more effective than an immediate trauma-focused approach in people with childhood-trauma related PTSD (Netherlands Trial Registry no.: NTR5991).

**Method:**

Adults with PTSD following childhood abuse were randomly assigned to either a phase-based treatment condition (8 sessions of Skills Training in Affect and Interpersonal Regulation (STAIR), followed by 16 sessions of eye-movement desensitisation and reprocessing (EMDR) therapy; *n* = 57) or an immediately trauma-focused treatment condition (16 sessions of EMDR therapy; *n* = 64). Participants were assessed for symptoms of PTSD and complex PTSD, and other forms of psychopathology before, during and after treatment and at 3- and 6-month follow-ups.

**Results:**

Data were analysed with linear mixed models. No significant differences between the two treatments on any variable at post-treatment or follow-up were found. Post-treatment, 68.8% no longer met PTSD diagnostic criteria. Self-reported PTSD symptoms significantly decreased for both STAIR–EMDR therapy (*d* = 0.93) and EMDR therapy (*d* = 1.54) from pre- to post-treatment assessment, without significant difference between the two conditions. No differences in drop-out rates between the conditions were found (STAIR–EMDR 22.8% *v.* EMDR 17.2%). No study-related adverse events occurred.

**Conclusions:**

This study provides compelling support for the use of EMDR therapy alone for the treatment of PTSD due to childhood abuse as opposed to needing any preparatory intervention.

Repeated trauma in childhood has been found to be a risk factor for developing complex post-traumatic stress disorder (complex PTSD).^1^ Complex PTSD was officially introduced in ICD-11 as part the ‘Disorders specifically associated with stress’ category.^2^ To fulfil the diagnostic criteria a person needs to display, in addition to PTSD criteria, disturbances in three domains of self-organisation (i.e. affect dysregulation, negative core beliefs and interpersonal problems). Eye-movement desensitisation and reprocessing (EMDR) therapy and prolonged exposure therapies are effective trauma-focused treatments for PTSD,^1^ but it is still unclear whether people who meet criteria for complex PTSD need a different treatment approach than those based on existing international treatment guidelines for PTSD.^3^

## Phase-based treatment approach

One of the treatment approaches for complex PTSD suggested, even before complex PTSD was included in the ICD-11, is a phase-based treatment.^4^ In the first phase – cognitive–behavioural therapy – patients experiment with emotion regulation and interpersonal skills. The second phase is trauma-focused treatment in which the traumatic memories are processed. The third and final phase focuses on the consolidation of treatment gains and the resumption of daily activities.^4^ The purpose of the first phase is also to establish a therapeutic relationship and increase safety and flexibility in skills.^5^

## Evidence for the phase-based approach

The value of using a phase-based approach was supported by the results of a three-armed randomised controlled clinical trial conducted by Cloitre et al.^6^ This study evaluated the effectiveness of a phased-based treatment approach by comparing three treatment conditions: (a) eight sessions of Skills Training in Affect and Interpersonal Regulation (STAIR),^5^ followed by eight sessions of prolonged exposure therapy; (b) supportive counselling followed by a comparable number of sessions of exposure therapy; and (c) STAIR followed by supportive counselling (*n* = 104). Because the outcomes of both conditions differed at follow-up for some variables (PTSD symptoms, interpersonal problems and several scales for affect regulation) in favour of the STAIR/exposure condition, the researchers concluded that the results of their study suggested that a phase-based approach is superior to a trauma-focused approach. However, the fact that a pure exposure condition was lacking makes it difficult to draw conclusions about the relative benefits of a phased-treatment approach over conventional trauma-focused PTSD treatment.^3^

## Comparison of the phased-based approach with the trauma-focused treatment

The most recent version of the treatment guidelines for complex PTSD published by the International Society of Traumatic Stress Studies (ISTSS) proposes a ‘personalised medicine’ approach aimed at identifying symptoms that are clinically significant to a particular patient and tailoring interventions to address these.^1^ The guideline committee acknowledged that research supporting this guideline is lacking and that only a head-to-head comparison of a phase-based treatment with an immediately trauma-focused treatment using a randomised controlled design can answer this important question. Recently, Oprel and her colleagues conducted a randomised controlled trial (RCT) in which prolonged exposure therapy was directly compared with a phase-based treatment (prolonged exposure therapy preceded by STAIR) among 149 individuals with PTSD due to childhood abuse.^7^ The hypothesis that the phase-based treatment would be associated with larger PTSD symptom reductions compared with the immediate prolonged exposure therapy was not supported by their data. A comparable study with EMDR therapy as the trauma-focused treatment has not been performed.

## Aim of the present study

The purpose of the present study was to help find an answer to the question as to whether a phase-based treatment approach is more effective than an immediately trauma-focused treatment in ameliorating the treatment outcomes in individuals with PTSD due to repeated sexual and/or physical childhood abuse. To make a direct comparison between both treatment options, two types of therapy were applied: EMDR therapy preceded by STAIR,^5^ and immediate EMDR therapy not preceded by STAIR. Because STAIR was developed especially for individuals with PTSD and symptoms of complex PTSD to ameliorate treatment outcomes, we hypothesised that, compared with the EMDR therapy only condition, the phase-based treatment condition would be significantly more effective in reducing symptoms of PTSD, symptoms of complex PTSD and other forms of psychopathology, and would also lead to significantly less drop-out than the stand-alone trauma-focused condition.

## Method

### Design

The study is a single-blind RCT with two arms (EMDR therapy versus EMDR therapy preceded by STAIR) with measurements at pre-treatment, every eight sessions, post-treatment and at 3- and 6-month follow-up. The design paper for this trial^8^ is available at https://doi.org/10.1186/s13063-018-2508-8. This also includes a CONSORT checklist. The study design was registered in the Netherlands Trial Registry (NTR5991) and approved by the medical ethics committee of the University of Twente, The Netherlands (56641.044.16 CCMO).

### Participants

Participants were recruited from two out-patient mental health organisations in The Netherlands (Dimence and GGZ Oost-Brabant) from 5 September 2016, and the last follow-up assessment was on 28 August 2020. Patients were included if aged between 18 and 65 years and diagnosed with PTSD as measured by the Clinician-Administered PTSD scale for DSM-5 (CAPS-5).^9^ Furthermore, they had to be a victim of repeated sexual and/or physical abuse before the age of 18 by a caretaker or a person in a position of authority, as identified by the LEC-5.^10^

### Exclusion criteria

Patients were excluded if they displayed insufficient Dutch language proficiency or an acute risk of suicidality for which immediate crisis intervention was needed, as assessed by item 9 of the Beck Depression Inventory - II (BDI-II).^11^ Also, they were excluded if they had received treatment for PTSD in the past year with at least eight sessions (any well-evaluated programme), reported being a victim of ongoing physical and/or sexual abuse, reported alcohol or drug dependence or misuse according to DSM-5 criteria^12^ during screening for eligibility or if they had an intellectual disability at registration at the institution.

### Procedure

Individuals eligible for inclusion received oral and written information about the study. If they agreed to participate and signed the informed consent form, they were assessed for inclusion (*n* = 151). After inclusion, participants were randomly assigned to one of the two treatment conditions (the exact randomisation procedure is described in the design paper^8^).

### Measurements

The self-reported severity of PTSD symptoms was the primary outcome and was assessed by the PTSD Symptom Scale – Self-Report version (PSS-SR)^13^ at pre-treatment, after eight sessions and at post-treatment. The internal reliability at baseline was high (Cronbach's *α* = 0.83).

Several secondary outcome measures were included. The presence and severity of PTSD diagnoses were assessed using the CAPS-5^9^ at pre-treatment, post-treatment and at both follow-ups.

The presence and severity of symptoms of complex PTSD were measured using the Structured Interview for Disorders of Extreme Stress (SIDES),^14^ more specifically the 38-item version developed by Ford et al.^15^

We investigated distinct symptoms of complex PTSD pre-treatment, after eight sessions and post-treatment as follows. In addition to the PTSD symptoms as indexed by the CAPS,^9^ symptoms of complex PTSD were measured based on the symptom clusters of the ICD-11 complex PTSD classification,^2^ that is, by using the Inventory of Interpersonal Problems (IIP)^16^ to measure interpersonal difficulties (Cronbach's α = 0.85 at baseline of the current study), the Difficulties in Emotion Regulation Scale (DERS)^17^ to assess difficulties in emotion regulation (Cronbach's α = 0.92 at baseline of the current study) and the Posttraumatic Cognitions Inventory (PTCI)^18^ to index trauma-related thoughts and beliefs (Cronbach's α = 0.96 at baseline of the current study).

Trait dissociation was measured using the Dissociative Experiences Scale II (DES-II)^19^ (Cronbach's *α* = 0.93 at baseline of the current study) at pre-treatment, post-treatment and both follow-ups.

The Brief Symptom Inventory^20^ (Cronbach's *α* = 0.95 at baseline of the current study) was used to index symptoms of general psychopathology at every measurement point.

### Treatment

In both treatment conditions, participants first received one 90 min session, consisting of psychoeducation and determining a hierarchy of relevant traumatic experiences. Both the STAIR and EMDR therapy were thereafter delivered twice a week for 90 min and sessions were video recorded. STAIR was conducted according to the programme described by Cloitre and her colleagues.^5^ EMDR therapy was conducted according to the protocol by Shapiro using the Dutch translation of the treatment protocol.^21^ After treatment (8 sessions of STAIR followed by 16 sessions of EMDR therapy, or 16 sessions of EMDR therapy only), participants were not allowed to receive psychological therapy for 6 months. Psychiatric consultation or support by a nurse were allowed to prevent deterioration during treatment and follow-up, but this had to be reported. Participants were considered to have dropped out of the study if they left any time after the first session. Patients were considered as early completers if all their trauma targets were processed and they did not meet the criteria for PTSD anymore before the maximum amount of treatment sessions. Those who dropped out as well as early completers were assessed at the planned time points. Regular monitoring of participant safety was conducted by therapists and researchers, and serious adverse events were recorded and reported to the main researcher, who reported these to the medical ethics committee.

### Therapists

All 27 participating therapists were experienced psychologists and already trained (at advanced level) in EMDR therapy prior to the trial. In addition, they received 2 days of training in STAIR from an experienced STAIR protocol trainer (M.-L.M.) and a half-day training on how to make a hierarchy of relevant traumatic experiences with the patient from another of the authors (A.d.J.). Within each condition, the participants were assigned to a therapist based on availability.

### Supervision and treatment fidelity

Supervisions took place every 2 months for both EMDR therapy (provided by an EMDR Europe accredited trainer, A.d.J.) and STAIR (provided by an experienced STAIR protocol trainer, M.-L.M). All therapists received individual feedback about the first set of video-recorded sessions at the start of a treatment. For the rating of treatment fidelity, 15% of the sessions were randomly selected and rated by the researchers for treatment adherence. A perfectly executed STAIR protocol was rated as 100% (the percentage of well-performed interventions per session), and the mean percentage over the rated sessions was 98.01% (s.d. = 6.04). For EMDR therapy sessions the maximum number of rated points was 15 (one point for every observed step of the protocol), and the mean number over the rated sessions was 14.45 (s.d. *=* 0.69). For each treatment condition, six recorded sessions were rated by a second independent rater, in order to compute the level of agreement between the researchers and the rater. The agreement between the researcher and the independent rater was 92.5% in the STAIR–EMDR condition and 96.7% in the EMDR condition and inconsistencies were discussed.

### Statistical analysis

Analyses were conducted using SPSS 27 for Windows.^22^ Possible demographic baseline differences were analysed using χ^2^-tests. Analyses were performed on an intention-to-treat basis. For dichotomous outcomes, a χ^2^-test was used to test differences in outcome between the treatment conditions. For continuous variables, we used a linear mixed models (LMM) analysis, with treatment conditions and time (i.e. measurements at different time points) as categorical variables, using a covariance pattern model.^23^ Compound symmetry was used as the covariance type and the standard restricted maximum likelihood (REML) as the estimation method. The STAIR–EMDR condition was the reference category. The analyses were conducted both with and without pre-measurement of the respective outcome variable as a covariate. As the mixed linear model analyses (LMM) without a covariate provides a better overview for complete changes over time, these are represented in the main text. The results for the LMM analyses with the pre-treatment as a covariate are presented Supplementary Tables 3a and 3b, available at https://doi.org/10.1192/bjo.2021.1057). To determine whether participants experienced symptom improvement beyond what could be attributed to measurement error, the reliable change index (RCI) for the CAPS was calculated^24^ in combination with test–retest reliability information based on previous research.^25^

### Ethics statement

The authors assert that all procedures contributing to this work comply with the ethical standards of the relevant national and institutional committees on human experimentation and with the Helsinki Declaration of 1975, as revised in 2008. All procedures involving human participants were approved by the Institutional Review Board of the University of Twente (merged with the Institutional Review Board United), reference number P16–03.

## Results

### Patient flow and sample characteristics

**Fig. 1:**
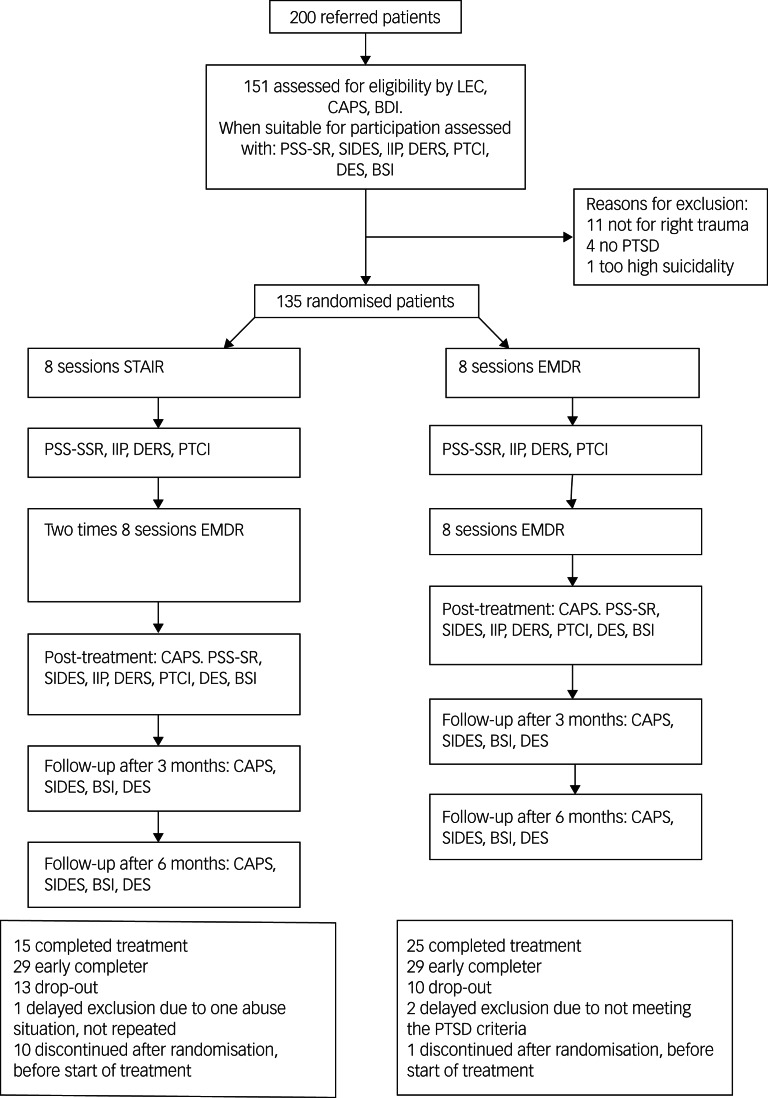
Flow of study participants. LEC, Life Events Checklist for DSM-5 (LEC-5); CAPS, Clinician-Administered PTSD scale for DSM-5; BDI, Beck Depression Inventory (BDI-II); PSS-SR, PTSD Symptom Scale – Self-Report version; SIDES, Structured Interview for Disorders of Extreme Stress; IIP, Inventory of Interpersonal Problems; DERS, Difficulties in Emotion Regulation Scale; PTCI, Posttraumatic Cognitions Inventory; DES, Dissociative Experiences Scale (DES-II); BSI, Brief Symptom Inventory; PTSD, post-traumatic stress disorder; STAIR, Skills Training in Affect and Interpersonal Regulation; EMDR, eye-movement desensitisation and reprocessing.

Figure 1 shows the flowchart of participants through the trial. Ten individuals in the STAIR–EMDR group and one in the EMDR group discontinued the trial before the start of treatment. The psychopathological and demographic characteristics of participants and those who discontinued did not differ at baseline, except for a significantly lower score on the self-report on the PTSD symptom scale in the group of patients who did not participate (*t*(118) = 2.20, *P* = 0.03). The demographic characteristics at baseline are presented in Supplementary Table 1, with no demographic differences between the treatment groups, except for living condition (situation) (with relatively more cohabitating participants in the EMDR group and more patients living alone in the STAIR–EMDR group). Given the RCT design we did not correct for pre-treatment differences.^26^

### Overall pre- to post-treatment effects

In both treatment groups, participants showed significant pre- to post-treatment reductions (i.e. a significant effect of time) on all variables (i.e. symptoms of PTSD, symptoms of complex PTSD and other forms of psychopathology; Supplementary Tables 2a and 2b). The within-group effect sizes pertaining to these differences were medium to large in both groupsfor the different variables (*d* = 0.50–1.70; Table 1).^27^ Based on the CAPS, 68.8% of all participants across treatment conditions did not fulfil the diagnostic criteria for PTSD at post-treatment. Based on the SIDES, only 3.3% of the participants still met the criteria for complex PTSD at post-treatment (compared with 28.9% prior to treatment).

**Table 1:** Mean assessment scores and between-group effect sizes at post-treatment, 8-week and 12-week follow-up, and within-group effect sizes^a^

	Pre-treatment		Between-group effect size post-treatment			Between-group effect size at 3-month follow-up			Between-group effect size at 6-month follow-up			Within-group effect size between pre- and post-treatment measurements	
	STAIR–EMDR, mean (s.d.)	EMDR, mean (s.d.)	STAIR–EMDR, mean (s.d.)	EMDR, mean (s.d.)	*d*	STAIR–EMDR, mean (s.d.)	EMDR, mean (s.d.)	*d*	STAIR–EMDR, mean (s.d.)	EMDR, mean (s.d.)	*d*	STAIR–EMDR	EMDR
PSS-SR	27.67 (8.23)	31.04 (9.11)	15.53 (11.71)	16.78 (11.75)	0.11	n.a.	n.a.		n.a.	n.a.		0.93	1.54
CAPS	37.61 (8.32)	39.34 (9.61)	17.44 (13.20)	19.04 (14.33)	0.12	14.50 (13.45)	16.02 (12.85)	0.12	14.24 (3.53)	16.20 (12.76)	0.15	1.70	1.66
SIDES-R	30.77 (10.96)	31.90 (14.03)	19.95 (13.48)	18.10 (11.81)	0.15	17.69 (13.42)	14.05 (10.72)	0.31	16.56 (14.62)	15.55 (11.44)	0.08	0.91	1.04
IIP	1.58 (0.54)	1.72 (0.61)	1.26 (0.74)	1.28 (0.73)	0.03	n.a.	n.a.		n.a.	n.a.		0.50	0.94
DERS	110.47 (21.78)	118.08 (27.35)	94.00 (25.79)	96.81 (27.79)	0.10	n.a.	n.a.		n.a.	n.a.		0.78	0.74
PTCI	130.76 (39.36)	142.89 (42.52)	99.70 (49.38)	102.71 (45.26)	0.06	n.a.	n.a.		n.a.	n.a.		0.87	0.85
DES	21.42 (13.16)	28.30 (20.16)	12.36 (14.73)	16.02 (13.41)	0.26	12.91 (14.91)	13.25 (13.74)	0.24	11.20 (14.22)	11.48 (11.10)	0.02	0.71	0.77
BSI	1.72 (0.68)	2.03 (0.78)	1.16 (0.83)	1.72 (0.86)	0.14	1.06 (0.90)	1.10 (0.73)	0.05	0.97 (0.89)	1.05 (0.71)	0.10	0.82	0.95

STAIR, Skills Training in Affect and Interpersonal Regulation; EMDR, eye-movement desensitisation and reprocessing; PSS-SR, PTSD Symptom Scale – Self-Report version; CAPS-5, Clinician Administered PTSD Scale for DSM-5; SIDES, Structured Interview for Disorders of Extreme Stress; IIP, Inventory of Interpersonal Problems; DERS, Difficulties in Emotion Regulation Scale; PTCI, Posttraumatic Cognitions Inventory; BSI, Brief Symptom Inventory; DES, Dissociative Experiences Scale (DES-II); n.a., not available.

a. Effect sizes are Cohen's *d*. Within-group effect size was calculated between pre-treatment and post-treatment.

### Treatment effectiveness of phase-based versus immediate trauma-focused treatment

None of the variables showed a significant effect of treatment condition or a significant treatment × time interaction effect from pre- to post-treatment (Supplementary Tables 2a and 2b).

**Fig. 2:**
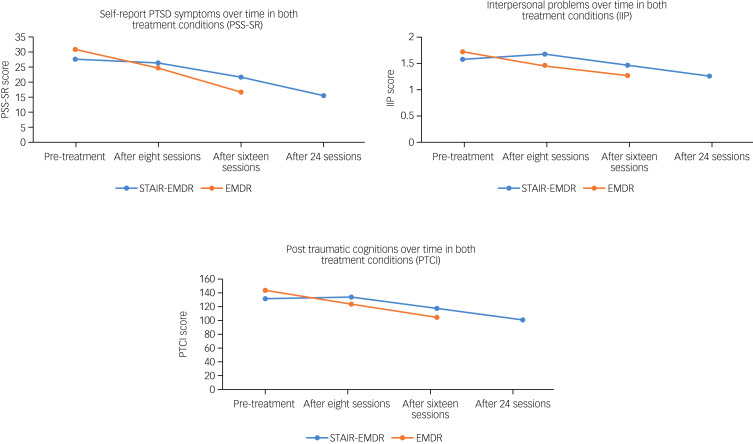
Mean scores pre-treatment and after 8, 16 and 24 sessions for the PTSD Severity Scale Self-Report (PSS-SR), the Inventory of Interpersonal Problems (IIP) and the Posttraumatic Cognitions Inventory (PTCI). PTSD, post-traumatic stress disorder; STAIR, Skills Training in Affect and Interpersonal Regulation; EMDR, eye-movement desensitisation and reprocessing.

For three variables, we did find a differential course of symptom decrease between pre- and post-treatment (i.e. although the levels were comparable at post-treatment; Supplementary Table 2a). This was the case for self-report of PTSD symptoms (PSS-SR; *F*(2, 164.26) = 3.90, *P* = 0.022), interpersonal problems (IIP; *F*(2, 155.17) = 4.86, *P* = 0.009) and post-traumatic cognitions (PTCI; *F*(2, 158.22) = 4.17, *P* = 0.017). As shown in Fig. 2, a comparable pattern was present for these three variables, as symptom decrease in the STAIR–EMDR group only began during the EMDR part (i.e. from eight sessions to post-treatment) and not in the STAIR part (i.e. the first eight sessions). In contrast, in the EMDR group the decline in symptoms was gradual across the sessions from pre- to post-treatment.

The dichotomous CAPS outcome did not show a significant difference in the proportions who no longer met PTSD diagnostic criteria between the two treatment groups at post-treatment (66.7% in the EMDR group and 68.9% in the STAIR–EMDR group; χ^2^(2, 96) = 0.05, *P* = 0.816). The proportion of participants who no longer met complex PTSD diagnostic criteria was 28.8% in the EMDR group and 22.2% in the STAIR–EMDR (pre-treatment, 32.8% met the complex PTSD diagnosis in the EMDR group and 24.6% in the STAIR–EMDR group). No significant differences between the proportion of individuals with a reliable change on the CAPS were found (RCI = 21.4; 53.3% in the STAIR–EMDR group and 45.1% in the EMDR group; χ^2^(1, 96) = 0.36, *P* = 0.55).

### Long-term treatment results

For the variables measured at follow-up (Supplementary Table 2b), no significant treatment effect, time effect or treatment × time interaction was found between post-treatment measurement and the 3-month follow-up. Also, no significant changes across to time were found between 3- and 6-month follow-up for scores on the CAPS (*t*(268.42) = 0.238, *P* = 0.812,) SIDES (*t*(251.12) = 0.90, *P* = 0.367), DES (*t*(233.63) = 1.21, *P* = 0.226) and BSI (*t*(244.00) = 0.821, *P* = 0.413).

### Early completion, serious adverse events and drop-out

The percentage of early completers (STAIR–EMDR group: 50.9%; EMDR group: 45.3%), participants who dropped out (STAIR–EMDR group: 22.8%; EMDR group: 17.2%) and participants who completed all sessions (STAIR–EMDR group: 26.3%; EMDR group: 37.5%) did not differ significantly between the two treatment groups at post-treatment (χ^2^(2, 121) = 1.85, *P* = 0.40). We did not find clinical or demographic characteristics related to drop-out. In the STAIR–EMDR group one serious non-study-related (as assessed by the medical ethics committee) adverse event was reported, which included short hospital admission after a suicide attempt. In the EMDR group two non-study-related adverse events were reported (one due to increased suicidal ideation during the follow-up and one due to increased psychotic experiences following changes in medication).

## Discussion

In contrast to our hypothesis, that phase-based treatment would lead to better treatment outcomes than stand-alone trauma-focused treatment, no differences were found between the two treatment groups on any variable indexing PTSD, complex PTSD or other forms of psychopathology. The only differences between the groups consisted of faster recovery (i.e. within eight sessions) in the EMDR therapy group on self-reported PTSD symptoms, interpersonal problems and post-traumatic cognitions, but this advantage did not last, as the results between groups were found to be comparable at later time intervals. Because the drop-out rates for both treatment groups did not differ significantly, the hypothesis that a phase-based treatment would lead to significantly less attrition than an immediate trauma-focused treatment could also not be supported. These results suggest that a stabilisation phase is not a necessary condition for applying a trauma-focused treatment in a sample of individuals with PTSD due to repeated sexual and/or physical childhood abuse. The results confirm the findings of other studies indicating that both phase-based and immediate trauma-focused therapies are safe and effective treatments (e.g.^28–30^).

This was the third RCT that has been conducted so far comparing a phase-based treatment with a trauma-focused treatment in people with PTSD due to childhood abuse,^6,7^ but the first using EMDR therapy. The results of this study are in line with both previous studies in that no differences were found between the treatment groups at post-treatment in favour of a phase-based treatment approach. In other words, both prolonged exposure therapy and EMDR therapy are likely to be safe and effective treatments for individuals suffering from symptoms of complex PTSD due to childhood abuse, and a stabilisation phase does not additionally improve treatment results. On the contrary, according to our results, for some symptoms (i.e. interpersonal problems, post-traumatic cognitions), a phase-based treatment approach may even delay the recovery process.

The present study also found no support for the hypothesis that EMDR therapy preceded by STAIR would lead to less drop-out than EMDR only. To this end, the results are in line with the results of Oprel et al,^7^ but in contrast to the study of Cloitre et al,^6^ which showed that significantly fewer people dropped out of the STAIR/exposure group compared with the support/exposure group. However, a closer look at this study reveals that the higher drop-out rate in the support/exposure group was largest during the supportive counselling phase, not during the trauma-focused part.

### Strengths and limitations

The present study has several strengths. Most important, the treatments were performed in two regular psychiatric out-patient settings. This increases the generalisability of these research findings. Another strength is that therapists received individual feedback on their treatment sessions, which may have led to treatment efficiency and high levels of treatment integrity. Conversely, a limitation of the study is that the two treatment groups were not compared with an inactive control group controlling for effects resulting from the mere passage of time. A second critical note refers to the fact that STAIR was offered twice a week, instead of once a week; this may have led to less time to practise new skills in between sessions.

### Future research

Despite the compelling support for the use of EMDR therapy alone for the treatment of complex PTSD as opposed to needing any preparatory intervention it remains important to investigate in future studies whether specific symptoms of complex PTSD, and maybe also other relevant patient characteristics, might moderate treatment outcome in that some patients might benefit more from an immediately trauma-focused treatment whereas others may benefit more using a phase-based approach. More specifically, because not everyone fully benefited from EMDR therapy alone (25% still fulfilled the diagnostic criteria for PTSD), in future research it seems sensible and important to explore which variables determine an adequate or less adequate treatment response, and whether there may still be patients who benefit from some form of emotion regulation or other skills training. In addition, future studies should investigate the long-term effects of different trauma treatments, as the course of separate symptoms during follow-up over the long term may differ between the different treatments.

### Clinical and management implications

Our findings, and those of other recent studies on the treatment of symptoms of complex PTSD in relation to the phase-based approach, fit into a new vision that is emerging on this topic; that is, that a phase-based treatment for symptoms of complex PTSD is effective, but not necessary. As the duration of therapy is much longer and needs more resources in the phase-based condition, this suggests that time and finances for commitment to treatment of patients as well as training and supervision of therapists may be better spent by focusing on single trauma-focused interventions.

## Data Availability

The data that support the findings of this study are available on request from the corresponding author.

## Supplementary material

For supplementary material accompanying this paper visit http://dx.doi.org/10.1192/bjo.2021.1057. click here to view supplementary material
