# Supplementary material for: Phase-based treatment versus immediate trauma-focused treatment for post-traumatic stress disorder due to childhood abuse: randomised clinical trial
Source: BJPsych Open. 2021 Nov 16;7(6):e211. doi: 10.1192/bjo.2021.1057 (PMC8612023; doi:10.1192/bjo.2021.1057)
Supplement: Supplementary file 1 [file bjosup.zip › S2056472421010577sup001.docx]

Supplemental Table 1: Demographic characteristics at baseline

| Characteristic | STAIR-EMDR  (n=57)  % of total population | EMDR  (n=64)  % of total population | Total sample  (n=121) | ꭓ^2^ | df | *p* |
| --- | --- | --- | --- | --- | --- | --- |
| Gender |  |  |  | 0.03 | 1 | .88 |
| Male | 14.0% (n = 17) | 17.4% (n = 21) | 31.4% (n = 38) |  |  |  |
| Female | 33.1% (n = 40) | 35.5% (n = 43) | 68.6% (n = 83) |  |  |  |
| Education |  |  |  | 2.94 | 2 | .23 |
| Low | 25.9% (n = 30) | 19.8% (n = 23) | 45.7% (n = 53) |  |  |  |
| Middle | 16.4% (n = 19) | 25,0% (n = 29) | 41,4% (n = 48) |  |  |  |
| High | 6.0% (n = 7) | 6.9% (n = 8) | 12.9% (n = 15) |  |  |  |
| Employment |  |  |  | 0.04 | 2 | .98 |
| Unemployed | 28.6% (n = 34) | 31.9% (n = 38) | 60.5% (n = 72) |  |  |  |
| Employed | 13,4% (n = 16) | 14,3% (n = 17) | 27,7% (n = 33) |  |  |  |
| Student | 5.9% (n = 7) | 5.9% (n = 7) | 11.8% (n = 14) |  |  |  |
| Living Condition |  |  |  | 8.40 | 3 | .04 |
| Married or cohabitating | 14.4% (n = 17) | 30.5% (n = 36) | 44.9% (n = 53) |  |  |  |
| Alone | 23.7% (n = 28) | 16.9% (n = 20) | 40.7% (n = 48) |  |  |  |
| With relatives or friends | 5.1% (n = 6) | 4.2% (n = 5) | 9.3% (n = 11) |  |  |  |
| Sheltered house | 3.4% (n = 4) | 1.7% (n = 2) | 5.1% (n = 6) |  |  |  |
| Sexual abuse (% per condition) | 37.2% (n = 45) | 37.2% (n = 45) | 74.4% (n = 90) | 0.77 | 1 | .38 |
| Physical abuse (% per condition) | 37.2% (n = 45) | 41.3% (n = 50) | 78.5% (n = 95) | 0.00 | 1 | 1.00 |
| Dissociative subtype (% per condition) | 14.0% (n = 17) | 20.7% (n = 25) | 34.7% (n = 42) | 0.76 | 1 | .38 |
| Suicidality |  |  |  | 0.81 | 2 | .67 |
| No suicidal thoughts | 10.7% (n = 13) | 14% (n = 17) | 24.8% (n =30) |  |  |  |
| Suicidal thoughts, no  intention | 28.1% (n = 34) | 27.3% (n = 33) | 55.4% (n = 67) |  |  |  |
| Preferring to cause  suicide | 8.3% (n = 10) | 11.6% (n = 14) | 19.8% (n = 24) |  |  |  |

Table 2a

Results of the Linear Mixed Model (LMM) of the primary and secondary outcome variables measured at pre-treatment, after eight sessions and at post-treatment.

|  | Treatment | | | Time | | | | | | | | | Treatment time interaction | | | | | | | | |
| --- | --- | --- | --- | --- | --- | --- | --- | --- | --- | --- | --- | --- | --- | --- | --- | --- | --- | --- | --- | --- | --- |
|  |  |  |  | Pre-treatment vs. post-treatment | | | Pre-treatment vs. after eight sessions | | | After eight sessions vs. post-treatment | | | Pre-treatment vs. post-treatment | | | Pre-treatment vs. after eight sessions | | | After eight sessions vs. post-treatment | | |
|  | Est | SE | 95%CI | Est | SE | 95% CI | Est | SE | 95%CI | Est | SE | 95% CI | Est | SE | 95%CI | Est | SE | 95% CI | Est | SE | 95%CI |
| PSS-SR | 3.87 | 1.96 | -0.01 7.74 | -11.78^***^ | 1.60 | -14.94 -8.63 | -0.52 | 1.68 | -3.83 2.79 | -11.27^***^ | 1.82 | -14.87 -7.67 | -2.84 | 2.18 | -7.15 1.47 | -6.67^**^ | 2.40 | -11.40 -1.94 | 3.83 | 2.51 | -1.13  8.79 |
| IIP | 0.16 | 0.12 | -0.07 0.40 | -0.31^***^ | 0.78 | -0.46 -0.16 | 0.16^*^ | 0.08 | 0.00 0.33 | -0.47^***^ | 0.89 | -0.65 -0.30 | -0.10 | 0.11 | -0.31 0.11 | -0.36^**^ | 0.12 | -0.59 -0.13 | 0.26^*^ | 0.12 | 0.02 0.50 |
| DERS | 7.21 | 4.74 | -2.15 16.57 | -15.49^***^ | 3.25 | -21.91 -9.08 | -1.90 | 3.42 | -8.66 4.85 | -13.59^***^ | 3.72 | -20.94 -6.24 | -3.31 | 4.52 | -12.24 5.63 | -5.40 | 4.92 | -15.11 4.32 | 2.09 | 5.13 | -8.04 12.22 |
| PTCI | 12.58 | 8.18 | -3.55 28.72 | -32.50^***^ | 5.84 | -44.03 -20.97 | 6.11 | 6,29 | -6.32 18.54 | -38.61^***^ | 6.75 | -51.95 -25.27 | -5.67 | 8.06 | -21.59 10.25 | -25.16^**^ | 8.85 | -42.63 -7.69 | 19.49^*^ | 9.21 | 1.31  37.67 |

Est = Estimated effect, SE = Standard Error, CI = Confidence Interval, PSS-SR = PTSD Symptoms Scale-self report, IIP = Inventory of Interpersonal Problems, DERS = Difficulties in Emotion Regulation Scale, PTCI = Posttraumatic Cognitions Inventory

*Note*. **p* < .05, ** p < . 01, ****p* < .001

Table 2b

Results of the Linear Mixed Model (LMM) for all outcome variables at pre-treatment, post-treatment and at

three- month’s follow-up*.*

|  | **Treatment** | | | **Time** | | | | | | **Treatment-time interaction** | | | | | |
| --- | --- | --- | --- | --- | --- | --- | --- | --- | --- | --- | --- | --- | --- | --- | --- |
|  |  |  |  | Pre- vs. post-treatment | | | Post-treatment vs. three month Follow-Up | | | Pre- vs. post-treatment | | | Post-treatment vs. three month Follow-Up | | |
|  | Est | SE | 95% CI | Est | SE | 95% CI | Est | SE | 95% CI | Est | SE | 95% CI | Est | SE | 95% CI |
| **CAPS-5** | 1.05 | 2.36 | -3.61 5.71 | -20.12^***^ | 1.62 | -23.30 -16.94 | -1.99 | 1.74 | -5.42 1.44 | -0.68 | 2.22 | -5.05 3.69 | 0.47 | 2.42 | -4.28 5.23 |
| **SIDES-R** | 1.63 | 2.53 | -6.62 3.35 | -11.14^***^ | 1.68 | -14.45 -7.82 | -0.69 | 1.80 | -4.23 2.85 | -2.32 | 2.30 | -6.85 2.20 | -2.10 | 2.48 | -6.99 2.79 |
| **BSI** | 0.16 | 0.16 | -0.15 0.47 | -0.53^***^ | 0.99 | -0.72 -0.33 | -0.06 | 0.11 | -0.27 0.15 | -0.18 | 0.14 | -0.45 0.09 | -0.08 | 0.15 | -0.37 0.20 |
| **DES** | 3.45 | 3.02 | -2.51 9.41 | -8.13^***^ | 1.95 | -11.96 -4.29 | 1.39 | 2.11 | -2.77 5.55 | -3.44 | 2.69 | -8.73 1.85 | -3.17 | 2.84 | -8.75 2.42 |

Est = Estimated effect, SE = Standard Error, CI = Confidence Interval, CAPS-5 = Clinician Administered PTSD Scale for DSM-5,

SIDES = Structured Interview for Disorders of Extreme Stress-Revised, BSI = Brief Symptom Inventory, DES = Dissociative Experiences Scale.

*Note.* **p* < .05, ** p < . 01, ****p* < .001

Supplemental Table 3a

Results of the Linear Mixed Model (LMM) for the primary and secondary outcome variables measured at pre-treatment, after eight sessions and at post-treatment, with the pre-treatment measurement as covariate.

|  | Treatment | | | Time | | | Treatment-time interaction | | |
| --- | --- | --- | --- | --- | --- | --- | --- | --- | --- |
|  |  |  |  | After eight sessions | | | After eight sessions | | |
|  | Est | SE | 95% CI | Est | SE | 95% CI | Est | SE | 95% CI |
| PSS-SR | -2.27 | 2.25 | -6.72  2.19 | 11.35^***^ | 2.00 | 7.37  15.34 | -4.20 | 2.80 | -9.78  -1.39 |
| IIP | -0.94 | 0.11 | -0.30  0.12 | 0.47^***^ | 0.10 | 0.28  0.66 | -0.23 | 0.13 | -0.50  0.04 |
| PTCI | -0.96 | 7.92 | -16.63  14.71 | 39.56^***^ | 6.77 | 26.07  53.05 | -21.47^*^ | 9.33 | -40.09  -2.86 |
| DERS | -0.25 | 4.54 | -9.24  8.75 | 13.83*** | 3.34 | 7.15  20.51 | -3.15 | 4.71 | -12.57  6.27 |

Est = Estimated effect, SE = Standard Error, CI = Confidence Interval, PSS-SR = PTSD Symptoms Scale-self report, IIP = Inventory of Interpersonal Problems, DERS = Difficulties in Emotion Regulation Scale, PTCI = Posttraumatic Cognitions Inventor

*Note*. **p* < .05, ** p < . 01, ****p* < .001

Supplemental Table 3b

Results of the Linear Mixed Model (LMM) for the outcome variables measured at pre-treatment, post-treatment and at three- and six month’s follow-up, with the pre-treatment measurement as covariate*.*

|  | Treatment | | | Time | | | | | | Treatment-time interaction | | | | | |
| --- | --- | --- | --- | --- | --- | --- | --- | --- | --- | --- | --- | --- | --- | --- | --- |
|  |  |  |  | Three month FU | | | Six month FU | | | Three month FU | | | Six month FU | | |
|  | Est | SE | 95% CI | Est | SE | 95% CI | Est | SE | 95% CI | Est | SE | 95% CI | Est | SE | 95% CI |
| CAPS-5 | -0.25 | 2.40 | -4.99 4.49 | -1.39 | 1.58 | -4.52 1.74 | -2.03 | 1.54 | -5.06 1.01 | 0.19 | 2.21 | -4.17 4.54 | 0.59 | 2.18 | -3.72 4.90 |
| SIDES | -1.97 | 2.27 | -6.45 2.52 | -0.67 | 1.54 | -3.72 2.38 | -2.97^*^ | 1.49 | -5.91 -0.02 | -1.94 | 2.15 | -6.18 2.30 | .81 | 2.11 | -3.36 4.99 |
| BSI | -0.10 | 0.15 | -0.27 0.50 | -0.05 | 0.09 | -0.22 0.12 | -0.16 | 0.83 | -0.33 0.00 | -0.06 | 0.12 | -0.29 0.18 | 0.53 | 0.12 | -0.18 0.29 |
| DES | -0.02 | 2.67 | -5.30 5.26 | 1.45 | 1.67 | -1.86. 4.75 | -1.17 | 1.61 | -4.35 2.01 | -3.03 | 2.30 | -7.57 1.51 | -1.76 | 2.30 | -6.31 2.78 |

Est = Estimated effect, SE = Standard Error, CI = Confidence Interval, CAPS-5 = Clinician Administered PTSD Scale for DSM-5, SIDES = Structured Interview for Disorders of Extreme Stress-Revised, BSI = Brief Symptom Inventory, DES = Dissociative Experiences Scale. For all variables the effect of the covariate was significant.

*Note.* **p* < .05, ** p < . 01, ****p* < .001
